# RF-PseU: A Random Forest Predictor for RNA Pseudouridine Sites

**DOI:** 10.3389/fbioe.2020.00134

**Published:** 2020-02-26

**Authors:** Zhibin Lv, Jun Zhang, Hui Ding, Quan Zou

**Affiliations:** ^1^Institute of Fundamental and Frontier Sciences, University of Electronic Science and Technology of China, Chengdu, China; ^2^Rehabilitation Department, Heilongjiang Province Land Reclamation Headquarters General Hospital, Harbin, China; ^3^Center for Informational Biology, University of Electronic Science and Technology of China, Chengdu, China

**Keywords:** pseudouridine sites, light gradient boosting, random forest, machine learning, RNA

## Abstract

One of the ubiquitous chemical modifications in RNA, pseudouridine modification is crucial for various cellular biological and physiological processes. To gain more insight into the functional mechanisms involved, it is of fundamental importance to precisely identify pseudouridine sites in RNA. Several useful machine learning approaches have become available recently, with the increasing progress of next-generation sequencing technology; however, existing methods cannot predict sites with high accuracy. Thus, a more accurate predictor is required. In this study, a random forest-based predictor named RF-PseU is proposed for prediction of pseudouridylation sites. To optimize feature representation and obtain a better model, the light gradient boosting machine algorithm and incremental feature selection strategy were used to select the optimum feature space vector for training the random forest model RF-PseU. Compared with previous state-of-the-art predictors, the results on the same benchmark data sets of three species demonstrate that RF-PseU performs better overall. The integrated average leave-one-out cross-validation and independent testing accuracy scores were 71.4% and 74.7%, respectively, representing increments of 3.63% and 4.77% versus the best existing predictor. Moreover, the final RF-PseU model for prediction was built on leave-one-out cross-validation and provides a reliable and robust tool for identifying pseudouridine sites. A web server with a user-friendly interface is accessible at http://148.70.81.170:10228/rfpseu.

## Introduction

More than 150 types of chemical modification have been identified in cellular RNA, including adenosine methylation, cytosine modification, isomerization of uridine, and ribose modification (Boccaletto et al., 2018). These modifications have critical roles in cellular biological and physiological processes (Song and Yi, 2017). For instance, one of the most prevalent RNA modifications in eukaryotes, N^6^-methyladenosine (m6A), affects RNA stability (Wang et al., 2014), RNA-protein interaction (Liu et al., 2015b), RNA splicing and translation (Meyer and Jaffrey, 2014), the circadian clock (Fustin et al., 2013), immune response (Winkler et al., 2019), etc. Another widespread RNA modification is 5-methylcytosine (m5C), which has functions including preservation of the secondary structure of tRNA (Motorin and Helm, 2010), control of amino-acylation (Helm, 2006), codon identification and metabolic stability (Agris, 2008; Li et al., 2017). The pseudouridine modification is another common post-transcriptional modification in various living organisms (Zaringhalam and Papavasiliou, 2016). In 1951, pseudouridine was first identified, and experiments in 1960 revealed that it was abundant in tRNA and rRNA (Cohn, 1960). Pseudouridine results from an isomerization of uridine by breaking the glycosidic bond with 180° base rotation (Karijolich et al., 2015). This modification has been shown to have vital roles, for instance, in stabilizing RNA and in the stress response (Zhao and He, 2015; Cheng et al., 2019a; Wang et al., 2019b).

Although RNA pseudouridylation was discovered decades ago, the first transcriptome-wide RNA pseudouridylation map was not published until 2014, following the rapid development of next-generation sequencing technology (Goodwin et al., 2016). Carlile et al. (2014) developed the PseudoU-seq technology, which they used to identify more than 200 pseudouridylation sites in the regulated mRNA of yeast and human cells; in the same year, Schwartz et al. (2014) performed transcriptome-wide mapping using a similar protocol, finding more than 300 dynamic-regulated pseudouridine sites in non-coding RNA and mRNA. Li et al. (2015a) presented a chemical labeling method (CeU-Seq) that they used to pull down more than 2000 pseudouridine sites in human mRNA. Other RNA pseudouridylation sequencing protocols were also developed (Carlile et al., 2015).

As an alternative to costly and labor-intensive laboratory experiments, robust, swift, and inexpensive computational methods for RNA chemical modification prediction have emerged recently, owing to the increasing amount of data generated in this post-genomics era (Libbrecht and Noble, 2015). A large number of m6A (Chen et al., 2015, 2018a,b, 2019a; Zhou et al., 2016; Zhao et al., 2019; Zou et al., 2019) and m5C (Feng et al., 2016; Qiu et al., 2017; Li et al., 2018; Sabooh et al., 2018; Zhang et al., 2018; Yin et al., 2019) site predictors based on traditional machine learning and emerging deep learning algorithms have been proposed. However, few computational tools have been developed to predict pseudouridine sites. Li et al. (2015b) used a support vector machine (SVM) classifier to design a web server called PPUS for the identification of pseudouridine sites in *Saccharomyces cerevisiae* and *Homo sapiens*. Chen et al. (2016) constructed another SVM-based web server for pseudouridine site prediction, using the frequency composition of the nucleotides and pseudo K-tuple nucleotide composition (PseKNC) for feature representation. He et al. (2018) presented another model, PseUI, to identify pseudouridine sites in RNA sequences from three species (*H. sapiens*, *S. cerevisiae*, and *M. musculus*); this was an SVM-based model incorporating multiple feature-extraction technologies. Tahir et al. (2019) used convolutional neural networks to design a new predictor, iPseU-CNN; and Liu et al. (2019b) developed the eXtreme gradient boosting (XGboost) method for RNA pseudouridine site prediction (XG-PseU). Cross-validation scores for RNA pseudouridine site identification in the abovementioned three species showed the best accuracy for iPseU-CNN (66.9%) in *H. sapiens*, whereas XG-PseU and iPseU-CNN had the best accuracy (68.2%) in *S. cerevisiae*, and XG-PseU was the most accurate (72.0%) in *M. musculus*. According to independent testing scores, iPseU-CNN outperformed the other models, with 69.0% accuracy in *H. sapiens* and 73.6% accuracy in *S. cerevisiae*. Although the iPseU-CNN predictor had a high average cross-validation accuracy (68.9%) and independent testing accuracy (71.3%) scores, there was still room for improvement in comparison with some high-performing m6A site predictors (Chen et al., 2019a; Zou et al., 2019).

**FIGURE 1:**
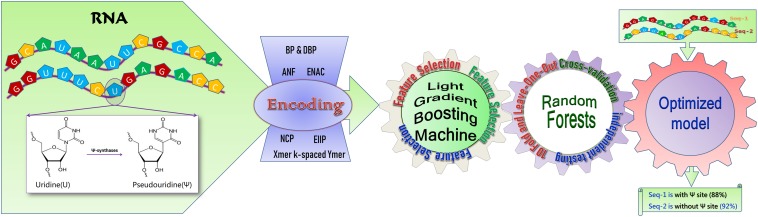
A schematic diagram of RF-PseU. RNA sequences with or without pseudouridine sites were encoded via seven RNA coding technologies; following removal of redundant features by light gradient boosting machine feature selection, the random forest model was trained on smaller but more relevant feature vector spaces, and was evaluated through cross-validation and independent testing to obtain an optimized model for prediction.

In this work, a model is developed based on the random forest algorithm, RF-PseU, for pseudouridine site recognition. The modeling overview is shown in Figure 1. RF-PseU incorporates multiple sequence feature representation technologies, and the light gradient boosting machine (LGBM) algorithm is employed to remove redundant features and rank the remaining features. Evaluation with leave-one-out (LOO) cross-validation demonstrated the robustness of the model. The average cross-validation accuracy (71.3% for 10-Fold and 71.4% for LOO) of RF-PseU was improved by 3.48–10.3% compared with existing state-of-the-art predictors, and the average independent testing accuracy (74.7%) showed a 4.8–19% increase. A user-friendly web server was also implemented, which can be accessed at http://148.70.81.170:10228/rfpseu. RF-PseU is expected to be a useful supplement to the existing tools for pseudouridine site identification.

## Materials and Methods

### Data Sets

Given that there were small differences between the benchmark data sets used in the studies of Chen et al. (2018a) and Liu et al. (2019b), data sets obtained from Chen et al. (2018a) were used to train and test our models. The training data sets included data for three species. That is, *H. sapiens* training dataset with 495 psedouridine-sites-containing sequences and 495 non-psedouridine-sites-containing; *S. cerevisiae* training dataset contains 314 psedouridine-sites-sequences and 314 non-psedouridine-sites-sequences; *M. musculus* training dataset consists of 944 sequences, half of which is positive samples. Whereas the independent testing data sets covered only two species, *H. sapiens* and *S. cerevisiae*, both of which contain 100 positive samples and 100 negative samples. For the *H. sapiens* and *M. musculus* data sets, the window size was 21, i.e. the positive samples were psedouridine site centroid sequences of 21 base pairs each, whereas those for the *S. cerevisiae* samples window site was 31, with psedouridine site centroid sequences of 31 base pairs. Negative samples, in which no psedouridine sites were detected, consisted of 21 base pairs for *H. sapiens* and *M. musculus*, and 31 base pairs for *S. cerevisiae*. The benchmark data sets can be downloaded from http://lin-group.cn/server/iRNAPseu/data.

### Feature Representation

Several widely used and convenient bio-sequence feature representation tools have been developed (Mrozek et al., 2013; Liu et al., 2015a, 2019c; Yu et al., 2015, 2019; Cheng and Hu, 2018; Hu et al., 2019; Muhammod et al., 2019). The two main tools used in this work were iLearn (Hu et al., 2019) and PyFeat (Muhammod et al., 2019).

#### Nucleotide Binary Profiles

Binary profiles encode the four bases (ACGU) as (1,0,0,0), (0,1,0,0), (0,0,1,0), and (0,0,0,1), whereas dibinary profiles encode the 16 dinucleotides (AA, AC, AG, AU, CA, CC, CG, CU, GA, GC, GG, GU, UA, UC, UG, and UU) as (0,0,0,0), (0,0,0,1), (0,0,1,0), (0,0,1,1),…, (1,1,1,1).

#### Accumulated Nucleotide Frequency

Suppose s_i_ is a base (ACGU) at the *i*^th^ position of a RNA sequence. Then we can determine the s_i_ density *d*_i_ of the *i*^th^ prefix subsequence of a RNA sequence as follows:

$$d_{i}=\frac{i}{\left|s_{i}\right|}\,\sum_{j=1}^{L}f\,\left(s_{i}\right),w\,h\,e\,r\,e\,f\,\left(q\right)=\{\begin{array}{c} 1,i\,f\,s_{i}=q\\ 0,o\,t\,h\,e\,r\,w\,i\,s\,e \end{array},$$

where *L* is the sequence length and *q* is one of the four nucleotides (ACGU).

#### Nucleotide Chemical Properties

The four RNA nucleotides (ACGU) are different from each other in terms of chemical structure and chemical bonds. On the basis of these differences, AGCU can be categorized into three different classes (Table 1) and encoded using a three-dimensional coordinate, i.e. A is denoted by (1,1,1), C by (0,1,0), G by (1,0,0), and U by (0,0,1).

**TABLE 1:** ACGU categories based on chemical properties.

**Nucleotides**	**Chemical property**
C,U	Pyrimidine and ring structure
A,G	Purine and ring structure
A,U	Weak and hydrogen bond
C,G	Strong and hydrogen bond
G,U	Keto and functional group
A,C	Amino and functional group

#### Electron-Ion Interaction Pseudopotentials (EIIP)

Nair and Sreenadhan (2006) used the EIIP values of A, G, C, and T (A: 0.1260, G: 0.0806, C: 0.1340, T: 0.1335) to directly represent the nucleotides in a DNA sequence. Here, iLearn was used to encode each nucleotide in the RNA sequences into EIIP feature vectors.

#### Enhanced Nucleic Acid Composition

The nucleotide composition was calculated for a fixed-length window of the RNA sequence, allowing the fixed window (length = 5) to continuously slide from the 5′ to the 3′ terminus. RNA sequences were then encoded into feature vectors of equal length.

#### Xmer k-Spaced Ymer Composition Frequency

This method is used to count the composition of a subsequence of X and Y consecutive nucleotides with intervals k, e.g. AGU@AU, A@CU, GU@@@A, where @ indicates a one-interval space, @@ a two-interval space, and so on. Generally, using Xmer k-spaced Ymer to encode an RNA sequence will generate a 4^X^ × 4^Y^ feature vector. In this study, X, Y, and k were set to 1, 2, or 3; and eight XYK combinations (except for 3mer-kspaced-3mer) were used for encoding. The PyFeat tool developed by Rafsanjani et al. (Muhammod et al., 2019) was used to convert RNA sequences into vectors.

### Feature Selection

Feature selection is an effective way to remove redundant information and prevent over-fitting in machine learning modeling (Tang et al., 2017; Xu et al., 2018a; Cheng et al., 2019a; Liu, 2019; Sun et al., 2019; Yu et al., 2019). Several feature selection technologies, including ANOVA (Lv et al., 2019b) and MRMD (Zou et al., 2016), have been developed and are widely used for DNA, RNA, and protein identification (Xu et al., 2018b). In this work, an LGBM (Ke et al., 2017)^1^ wrapper was used to select appropriate feature spaces for model training. In this process, raw training data were fed into the LGBM model and their features were ranked by importance value as calculated with the LGBM algorithm. Features with importance values greater than the average were selected to compose the feature space for modeling.

### Model Evaluation Metrics and Methods

The proposed models were evaluated by five commonly used metrics, accuracy (ACC), sensitivity (Sn), specificity (Sp), Matthew correlation coefficient (MCC), and integral area under the receiver operating characteristic curve (auROC). These metrics were calculated using the following equations, where TP, TN, FP, and FN stand for true positive, true negative, false positive, and false negative, respectively (Cheng et al., 2016, 2019b,c; Wei et al., 2017d, e; Liu et al., 2019a). For the ROC curve, 1-specificity was plotted on the horizontal axis, and sensitivity on the vertical axis.

$$\text{ACC}=\frac{T\,P+T\,N}{T\,P+T\,N+F\,P+F\,N}$$

$$S_{n}=\frac{T\,P}{F\,N+T\,P}$$

$$S_{n}=\frac{T\,N}{F\,P+T\,N}$$

$$\text{MCC}=\frac{T\,N\times T\,P-F\,N\times F\,P}{\sqrt{\left(T\,P+F\,P\right)\times \left(T\,P+F\,N\right)\times \left(T\,N+F\,N\right)\times \left(T\,N+F\,P\right)}}$$

LOO, K-Fold cross-validation, and independent testing are the most widely used methods for predictor evaluation (Mrozek et al., 2015; Cao and Cheng, 2016; Chen et al., 2017, 2018a, 2019b; Pan et al., 2017; He et al., 2018, 2019; Jiang et al., 2018; Xiong et al., 2018; Yu et al., 2018; Zhang et al., 2018; Ding et al., 2019; Feng et al., 2019; Kong and Zhang, 2019; Li and Liu, 2019; Lv et al., 2019a; Manavalan et al., 2019; Shan et al., 2019; Wang et al., 2019a; Wei et al., 2019a, b; Xu et al., 2019; Yu and Dai, 2019). That is the general machine learning evaluation methods (training, validation and testing) are used for optimized model evaluation. To test the efficiency of the classification, LOO cross-validation was performed for a data set containing n items, of which n-1 items were used for training and the remaining one was used for validation. This procedure was repeated until every sequence in the training data set had been used once as a validation testing sample. LOO cross-validation is robust but time-consuming for a large data set. To compare the performance of the model with that of existing predictors, 10-Fold cross-validation was also used. The training data set was stochastically divided into 10 subsets, with one subset for validation and the remaining nine for training. This process was repeated 10 times and the average results were used to evaluate the model. Finally, independent testing was performed to obtain a data set that was completely distinct from the training data set for evaluation of the trained model.

### Algorithm

The random forest method is a bagging-type ensemble learning algorithm (Cheng et al., 2018a, b). By combining multiple weak classifiers, the final results can be voted or averaged to obtain an overall model with higher accuracy, better general performance, and resistance to overfitting. This algorithm has been extensively used in bioinformatics and other areas, and has been confirmed to be an effective modeling technique in various domains (Ding et al., 2016a,b; Mrozek et al., 2016; Qiu et al., 2016; Wang et al., 2017; Wei et al., 2017a,b,c; Yu et al., 2017a; Zheng et al., 2017; Tang et al., 2018, 2019a; Xue et al., 2018; Degenhardt et al., 2019; Xu et al., 2019). In this study, the scikit-learn toolkit, available at https://scikit-learn.org, was used to establish the models.

Support vector machine (Cortes and Vapnik, 1995) is a generalized linear classifier that classifies data based on supervised learning; its decision boundary is the maximum-margin hyperplane required to solve the learning sample. SVM has been widely used in a variety of fields (Xiong et al., 2012; Ding et al., 2017; Yu et al., 2017b; Fu et al., 2018; Fang et al., 2019; Lai et al., 2019; Meng et al., 2019; Shen et al., 2019; Tang et al., 2019b; Zhang et al., 2019; Zhu et al., 2019). Here, it was used for modeling comparisons. SVM was also implemented via the scikit-learn toolkit, using the Gaussian radial basis functions, with the critical hyper-parameters (C and γ) of SVM optimized in a range from 10^–6^ to 10^6^ with exponent step 10^0.5^.

## Results and Discussion

### Optimization With Different Feature Spaces

**FIGURE 2:**
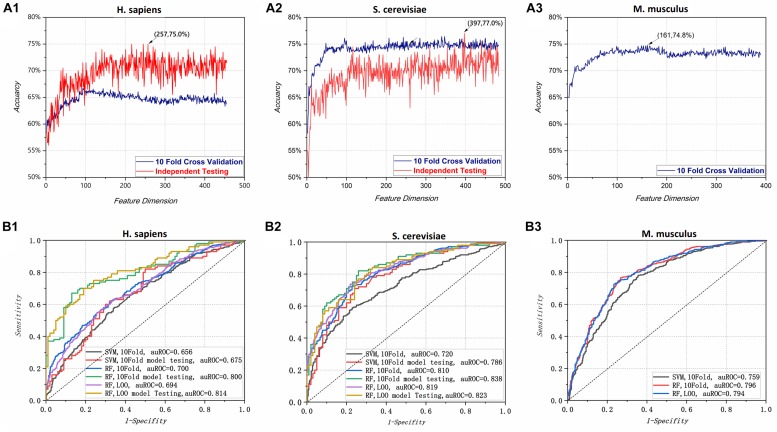
**(A)** Accuracy of the random forest predictor varied with feature dimension for all three species: **(A1)** *H. sapiens*; **(A2)** *S. cerevisiae*; **(A3)** *M. musculus*. The best independent accuracies for *H. sapiens* and *S. cerevisiae* were 75.0% with 257 features and 77.0% with 397 features, respectively, and the best 10-Fold cross-validated accuracy for *M. musculus* was 74.8% with 161 features. **(B)** Receiver operating characteristic curve (ROC) and area under the ROC curve (auROC) for different species under various conditions. **(B1)** is for *H. sapiens*, **(B2)** is for *S. cerevisiae* and **(B3)** is *M. musculus*. A support vector machine (SVM) was used for comparison with the random forest (RF) model. 10-Fold (10-Fold) model testing and leave-one-out (LOO) model testing indicate the model with the best 10-Fold and LOO cross-validation scores in independent testing. In cross-validation (10-Fold and LOO) and testing process, the training datasets have divided into training part and validation part. That is, they have used the general machine learning evaluation methods (training, validation and testing) for model optimization. In the figure, the 10-fold cross-validation and LOO cross-validation metric values are obtained from the validation part of training part, while the independent testing metric values are obtained from the independent testing datasets.

To determine optimal feature spaces, we first used the LGBM algorithm to sort the features from maximum to minimum according to their importance value. All the features with importance value greater than the average were kept. Second, we used an incremental feature selection strategy; as shown in Figure 2A, the 10-Fold cross-validation and independent testing accuracy varied as features were added. Initially, the accuracy increased rapidly for each species. As shown in Figure 2 (A1) and Figure 2 (A2), when the feature dimensions for *H. sapiens* and *S. cerevisiae* reached 257 and 397, the model achieved maximum independent testing accuracies of 75.0 and 77.0%, respectively. Owing to the lack of independent test data sets for *M. musculus*, Figure 2 (A3) shows only the cross-validation accuracy curve, with its peak value (74.8%) at a feature dimension of 161. The optimal feature space dimensions selected for each species were 257, 397, and 161, respectively. These values were used for further experiments and optimization.

### Comparison With SVM Predictors

Given that PPUS (Li et al., 2015b), iRNA-PseU (Chen et al., 2016), and PseUI (He et al., 2018) were all based on SVM, an optimized SVM model for pseudouridine site identification with the same feature spaces as the RF model was constructed to determine the effects of the SVM and RF on prediction performance. The performances of the two models are shown in Table 2. Overall, the models based on RF showed markedly better performance than those based on SVM. For instance, in terms of 10-Fold cross-validation accuracy, the RF models for *H. sapiens*, *S. cerevisiae*, and *M. musculus* outperformed the corresponding SVM models by 3.71%, 10.8%, and 5.80%, respectively. The independent testing accuracy scores showed an even greater contrast. For example, the RF model had 75.0% accuracy for *H. sapiens*, exactly 1.17 times that of the SVM model. The ROC curve and auROC value shown in Figure 2B also demonstrate that the optimized RF models performed better than the optimized SVM models for the same feature spaces. Thus, non-SVM models such as XG-PseU (Liu et al., 2019b), iPseU-CNN (Tahir et al., 2019), and our RF-PseU model might be more suitable for distinguishing pseudouridine sites from non- pseudouridine sites.

**TABLE 2:** Cross-validation and independent testing scores of two different classifiers for three species.

**Species**	**Algorithm**	**10 fold cross-validation**					**Independent testing**				
			
		**ACC**	**MCC**	**Sn**	**Sp**	**auROC**	**ACC**	**MCC**	**Sn**	**Sp**	**auROC**
*H. sapiens*	SVM	62.0%	0.240	61.4%	62.6%	0.656	64.0%	0.280	66.0%	62.0%	0.679
	RF	64.3%	0.287	66.1%	62.6%	0.700	75.0%	0.501	78.0%	72.0%	0.800
*S. cerevisiae*	SVM	67.5%	0.352	73.7%	61.2%	0.720	72.5%	0.45	73.0%	73.0%	0.786
	RF	74.8%	0.497	77.2%	72.4%	0.810	77.0%	0.540	75.0%	79.0%	0.838
*M. musculus*	SVM	70.7%	0.42	65.9%	75.4%	0.759	/	/	/	/	/
	RF	74.8%	0.50	73.1%	76.5%	0.796	/	/	/	/	/

### Comparison With Previous Predictors

The performance of RF-PseU was also compared with that of state-of-the-art predictors including iRNA-PseU (Chen et al., 2016), PseUI (He et al., 2018), iPseU-CNN (Tahir et al., 2019), and XG-PseU (Liu et al., 2019b). First, we compared the evaluation scores for the three species. Table 3 compares the cross-validation and independent testing scores for the state-of-the-art pseudouridine sites predictors with those of RF-PseU. In terms of cross-validation scores, the LOO accuracy values for *S. cerevisiae* and *M. musculus* were 75.4% and 74.5%, respectively, representing increments of approximately 10.5% and 3.47% over the values for the existing predictor (XG-PseU) with the best cross-validation score. However, the LOO accuracy of RF-PseU for *H. sapiens*, at 64.0%, showed a decrease of 4.0% compared with the best *H. sapiens* pseudouridine site predictor, PseU-CNN. In terms of independent testing, as shown in Table 3, RF-PseU scored higher than the existing predictors in all aspects. For comprehensive comparison, the average scores for different species were calculated. The results, shown in Table 4, demonstrate that RF-PseU performed better overall than the other four predictors. The cross-validation accuracy scores of RF-PseU were 3.48% higher than those of the best existing predictor, iPseU-CNN; in terms of independent testing scores, RF-PseU showed a marked improvement of 4.7–10.6% compared with iPseU-CNN. The overall performance of RF-PseU was also significantly better than those of the other predictors, indicating that RF-PseU can discriminate true pseudouridine sites from non-pseudouridine sites more precisely than the existing predictors.

**TABLE 3:** Comparison of cross-validation and independent testing scores of existing state-of-the-art pseudouridine site predictors and RF-PseU.

**Species**	**Classifier**	**Cross-validation**					**Independent testing**				
			
		**ACC**	**MCC**	**Sn**	**Sp**	**auROC**	**ACC**	**MCC**	**Sn**	**Sp**	**auROC**
*H. sapiens*	iRNA-PseU(LOO)^a^	60.4%	0.21	61.0%	59.8%	0.640	65.0%	0.30	60.0%	70.0%	/
	PseUI(LOO)^a^	64.2%	0.28	64.9%	63.6%	0.68	65.5%	0.31	63.0%	68.0%	/
	iPseU-CNN(5F)^b^	66.7%	0.34	65.0%	68.8%	/	69.0%	0.40	77.7%	60.8%	/
	XG-PseU (10F)^c^	66.1%	0.32	63.5%	68.7%	0.700	67.5%	/	/	/	/
	RF-PseU(10F)^d^	64.3%	0.29	66.1%	62.6%	0.700	75.0%	0.50	78.0%	72.0%	0.800
	RF-PseU(LOO)^e^	64.0%	0.29	65.9%	62.6%	0.694	74.0%	0.48	74.0%	74.0%	0.814
*S. cerevisiae*	iRNA-PseU(LOO)	64.5%	0.29	64.7%	64.3%	0.81	60.0%	0.20	63.0%	57.0%	/
	PseUI(LOO)	64.1%	0.30	64.7%	67.5%	0.69	68.5%	0.37	65.0%	72.0%	/
	iPseU-CNN(5F)	68.2%	0.37	66.4%	70.5%	/	73.5%	0.47	68.8%	77.8%	/
	XG-PseU(10F)	68.2%	0.37	66.8%	69.5%	0.77	71.0%	/	/	/	/
	RF-PseU(10F)	74.8%	0.49	77.2%	72.4%	0.810	77.0%	0.54	75.0%	79.0%	0.838
	RF-PseU(LOO)	75.8%	0.52	78.2%	73.4%	0.819	74.5%	0.49	70.0%	79.0%	0.823
*M. musculus*	iRNA-PseU(LOO)	69.1%	0.38	73.3%	64.8%	0.75	/	/	/	/	/
	PseUI(LOO)	70.4%	0.41	79.9%	70.3%	0.71	/	/	/	/	/
	iPseU-CNN(5F)	71.8%	0.44	74.8%	69.1%	/	/	/	/	/	/
	XG-PseU(10F)	72.0%	0.45	76.5%	67.6%	0.74	/	/	/	/	/
	RF-PseU(10F)	74.8%	0.50	73.1%	76.5%	0.796	/	/	/	/	/
	RF-PseU(LOO)	74.5%	0.48	72.7%	75.2%	0.794	/	/	/	/	/

*^*a*^Predictors based on support vector machine, Leave-One-Out Cross-Validation (LOO); ^*b*^Predictors based on convolutional neural nets, five-fold cross-validation (5F); ^*c*^Predictors based on XGboost,10-fold cross-validation (10F); ^*d*^Predictors based on Random Forest, 10-fold cross-validation; ^*e*^LOO:Leave-One-Out Cross-Validation*

**TABLE 4:** Comparison of average accuracies for state-of-the-art predictors.

**Scores type**	**RF-PseU (10 Fold^c^)**	**RF-PseU (LOO^d^)**	**iRNA-PseU (LOO)**	**PseUI (LOO)**	**iPseU-CNN (5 Fold^e^)**	**XG-PseU (10 Fold)**
Cross-validation^a^	71.3%	71.4%	64.7%	66.2%	68.9%	68.7%
Independent testing^b^	76.0%	74.7%	62.5%	67.0%	71.3%	69.3%

*^*a*^Average values of *H. sapiens*, *S. cerevisiae* and *M. musculus*; ^*b*^Average values of *H. sapiens* and *S. cerevisiae*; ^*c*^model with 10-fold cross-validation; ^*d*^model with leave-one-out cross-validation; ^*e*^model with five-fold cross-validation.*

### Web Server Implementation

**FIGURE 3:**
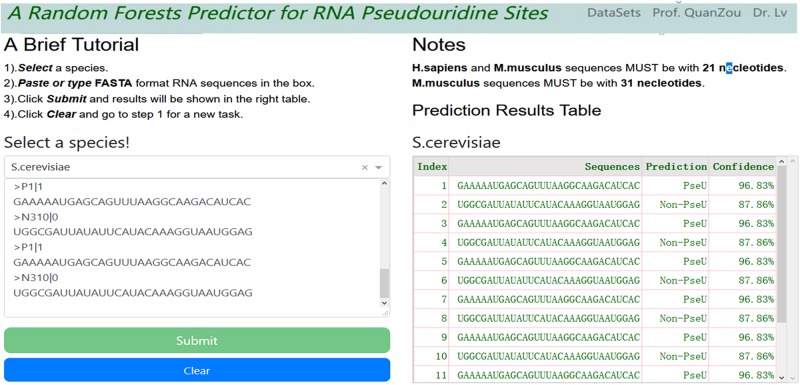
A screenshot of RF-PseU web server interface. The web server allows users to type or paste FASTA format text into the textbox and click submit button; the results are displayed in the right-hand table.

For convenience, a webserver with an easy-to-use interface was developed (see screenshot in Figure 3), which can be accessed freely at http://148.70.81.170:10228/rfpseu. A step-by-step user guide is given here. First, users select a species from the drop-down box and paste or type the query RNA sequences in FASTA format into the textbox. Second, after clicking the submit button, the query results will be shown in a table on the same page after a wait. Note that once a query task has been submitted, the submit button will be disabled. Third, the user can click the clear button to empty the input text box and enable the submit button, and return to step one to enter a new query task.

## Conclusion

In this study, a new model, named RF-PseU, for predicting RNA pseudouridine sites in multiple species is presented. For given feature spaces, the random forest algorithm was shown to be more efficient than SVM models for discriminating pseudouridine sites from non-pseudouridine sites. In terms of average cross-validation and independent testing accuracy scores, RF-PseU showed improvements of 3.6–10% and 4.8–21%, respectively, compared with state-of-the-art predictors. Moreover, a web server with a user-friendly interface is available. It is anticipated that RF-PseU will be a useful tool for RNA pseudouridine site analysis. However, the model requires further development via combination with other technologies before it is suitable for use as a classifier for RNA pseudouridine sites. Future work will explore emerging methods such as Gene2Vec (Zou et al., 2019), m6Acomet (Wu et al., 2019), and iterative feature representation (Wei et al., 2019b) to improve the model’s performance.

## Data Availability Statement

Publicly available datasets were analyzed in this study. This data can be found here: http://lin-group.cn/server/iRNAPseu/data.

## Author Contributions

ZL and JZ were responsible for experiments and manuscripts preparation. HD participated in discussions. QZ worked as supervisor for all procedures.

## Conflict of Interest

The authors declare that the research was conducted in the absence of any commercial or financial relationships that could be construed as a potential conflict of interest.

## Footnotes

1 https://lightgbm.readthedocs.io
